# Stratified treatment recommendation or one-size-fits-all? A health economic insight based on graphical exploration

**DOI:** 10.1007/s10198-018-1013-z

**Published:** 2018-10-29

**Authors:** Qi Cao, Erik Buskens, Hans L. Hillege, Tiny Jaarsma, Maarten Postma, Douwe Postmus

**Affiliations:** 1 Unit of PharmacoTherapy, -Epidemiology and -Economics, Groningen Research Institute of Pharmacy (GRIP), University of Groningen, Antonius Deusinglaan 1, 9713 AV Groningen, The Netherlands; 2 Department of Epidemiology, University of Groningen, University Medical Center Groningen, Hanzeplein 1, 9713 GZ Groningen, The Netherlands; 3 Department of Cardiology, University of Groningen, University Medical Center Groningen, Groningen, Hanzeplein 1, 9713 GZ Groningen, The Netherlands; 4 Department of Social and Welfare Studies, Faculty of Health Sciences, Linköping University, 581 83, Linköping, Sweden; 5 Institute for Science in Healthy Aging and healthcaRE (SHARE), University of Groningen, University Medical Center Groningen (UMCG), Hanzeplein 1, 9713 GZ Groningen, The Netherlands; 6 Department of Health Sciences, University of Groningen, University Medical Center Groningen (UMCG), Hanzeplein 1, 9713 GZ Groningen, The Netherlands

**Keywords:** Cost-effectiveness, Risk stratification, Personalized medicine, Heart failure, C02, C10, I10, I12

## Abstract

**Objectives:**

We sought to explore to what extent the use of Subpopulation Treatment Effect Pattern Plot (STEPP) may help to identify efficient treatment allocation strategy.

**Methods:**

The analysis was based on data from the COACH study, in which 1023 patients with heart failure were randomly assigned to three treatments: care-as-usual, basic support, and intensive support. First, using predicted 18-month mortality risk as the stratification basis, a suitable strategy for assigning different treatments to different risk groups of patients was developed. To that end, a graphical exploration of the difference in net monetary benefit (NMB) across treatment regimens and baseline risk was used. Next, the efficiency gains resulting from this proposed subgroup strategy were quantified by computing the difference in NMB between our stratified approach and the best performing population-wide strategy.

**Results:**

The analysis using STEPPs suggested that a differentiated approach, based on offering intensive support to low-risk patients (18-month mortality risk ≤ 0.16) and basic support to intermediate- to high-risk patients (18-month mortality risk > 0.16) would be an economically efficient treatment allocation strategy. This was confirmed in the subsequent cost-effectiveness analysis, where the average gain in NMB resulting from the proposed stratified approach compared to basic support for all was found to be €1312 (95% CI €390–€2346) per patient.

**Conclusions:**

STEPP provides a systematic approach to assess the interaction between baseline risk and the difference in NMB between competing interventions and to identify cutoffs to stratify patients in a health economically optimal manner.

## Introduction

Cost-effectiveness analysis supports resource allocation decision making by comparing the differences in costs and effects of alternative treatment regimens [1–4]. When such analyses are conducted alongside randomized controlled trials (RCTs), the cost-effectiveness of the evaluated treatments is generally expressed in terms of population averages. This provides insight into which of the available treatments performs best for the patient population considered. However, when these patients are characterized by a heterogeneous clinical condition, and their risk profiles are determined by factors like demographic variations, biometric variations, and co-morbidities, there may be considerable variation in response. In fact, the likelihood of subpopulations for whom response to one or the other treatment is obscured may be substantial [5–8]. Such differences among patients may also lead to systematic variation in resource use and costs, which could be another reason why one of the other treatments performs better in specific subpopulations [2, 6]. Acknowledging patient heterogeneity in health economic evaluation has therefore considerable potential in more efficient resource allocation decision-making [5, 8–10].

A recently conducted systematic review [5] identified baseline risk, treatment effect, health state utility, and resource utilization as the four input parameters of a health economic evaluation that may be prone to patient heterogeneity. However, as the cost-effectiveness of one treatment compared to another is ultimately determined by the net effect on all these parameters, it is essential that the impact of patient heterogeneity on each of these parameters is considered conjointly rather than in isolation, especially when the purpose is to identify more efficient reimbursement policies. For health economic evaluations conducted alongside an RCT, this can be achieved by conducting such analyses directly in terms of net monetary benefit (NMB) [11–13].

Hoch et al. [14] have previously proposed assessing the impact that different sources of patient heterogeneity may have on a treatment’s NMB by means of regression analysis. For example, suppose that one wants to explore whether the cost-effectiveness of a new treatment compared to the current standard treatment is affected by the age of the patient. Using regression analysis, this can be achieved by fitting a regression model with NMB as the dependent variable and the treatment indicator, age, and the interaction between age and the treatment indicator as the independent variables. A low *p* value for the regression coefficient corresponding to the interaction term then shows that age has a relatively strong influence on the new treatment’s relative cost-effectiveness.

While the use of multivariable regression models may provide insight into which sources of patient heterogeneity potentially have an impact on the relative cost-effectiveness of the evaluated treatments, the statistical power to detect such interaction effects is usually low. Moreover, actually being able to verify relevant heterogeneity using such models strongly depends on whether the assumed multiplicative structure of interaction fits reality. This may lead to missing or over-interpretation of the detected significant interaction terms. An alternative approach for studying treatment–covariate interaction that makes no assumptions about the nature of the relationship between the outcome and the covariate in each treatment group is the Subpopulation Treatment Effect Pattern Plot (STEPP) methodology [15–17]. This is based on a graphical exploration of the fluctuation in treatment effect across different, but overlapping subpopulations defined with respect to increasing levels of the covariate of interest. Although using STEPP to explore how the difference in NMB between two treatments varies as a function of one or more sources of patient heterogeneity could potentially be very useful in identifying more efficient reimbursement policies, to the best of our knowledge, it has not yet been considered. Using the difference in NMB as the measure of treatment benefit and an individualized predicted risk obtained from an RCT as the covariate of interest, the objective of this paper was to illustrate how the STEPP methodology can be used to derive risk-stratified treatment allocation strategies that maximize cost-effectiveness. Specifically, a case study in heart failure (HF) disease management was elaborated.

## Methods

### Study cohort

The data that we used to conduct our analysis was taken from the Coordinating study evaluating Outcomes of Advising and Counseling in Heart failure (COACH), a multicenter RCT in which 1023 patients were randomly assigned to one of three disease management programs (DMPs) [18, 19]. Patients in the care-as-usual group received routine follow-up management by a cardiologist. Along with this routine management, patients in the basic and intensive support groups received additional care from an HF nurse. In addition, patients in the intensive support group received multidisciplinary advice and two or more home visits by the HF nurse. The total follow-up time of the trial was 18 months.

### Baseline risk assessment

The patients’ predicted 18-month all-cause mortality risk was obtained from a previously developed multivariable risk prediction model [20]. This model included the following 14 predictor variables: age, gender, diastolic blood pressure, systolic blood pressure, history of stroke, history of myocardial infarction, atrial fibrillation, peripheral arterial disease, diabetes, left ventricular ejection fraction, previous HF hospitalization, serum sodium, estimated glomerular filtration rate (eGFR), and N-terminal pro brain natriuretic peptide (NT-proBNP). Missing values on these predictor variables were dealt with using multiple imputation [21]. Mortality risk values were then computed by taking the average of the risk values obtained from each of the ten imputed datasets.

### Patient-level NMB assessment

The patient-level NMB was calculated as ${\text{NM}}{{\text{B}}_i}=\lambda \times {e_i} - {c_i}$, where *e*_*i*_ and *c*_*i*_ denote the observed effect and cost for patient *i*, and *λ* denotes the willingness-to-pay threshold [14]. The patients’ observed survival time, which was censored at 18 months for those who were still alive at the end of the study’s follow-up, was taken as the measure of effectiveness. Costs were calculated at the patient level by multiplying the patients’ volumes of resource use with their respective unit costs as described in more detail in a previously conducted economic evaluation in this patient population [22]. The willingness-to-pay threshold was set equal to €20,000 per life year, which is the same threshold as was used in the aforementioned study [22].

### Exploration of treatment-predicted risk interaction and determination of subgroup strategy

To explore whether an interaction existed between the COACH DMPs and the predicted 18-month mortality risk, we applied the STEPP methodology [15, 16] using the difference in NMB as the outcome of interest. STEPP is a novel graphical method for assessing treatment–covariate interaction on different, but overlapping subpopulations defined with respect to the covariate of interest. The subpopulations are defined on the basis of two parameters: (1) the number of patients in common among consecutive subpopulations (*n*_1_) and (2) the number of patients in each subpopulation (*n*_2_). As the estimation of the interaction effect varies for different combinations of the parameter values, it is recommended to repeat the analysis for different values of n_1_ and n_2_ until the interaction effect stabilizes (i.e., a similar pattern is shown in the graph) [23]. For the analysis conducted in this study, stable estimates of the interaction effects were obtained by setting *n*_1_ and *n*_2_ equal to 120 and 150, respectively. Specifically, this means that the first subpopulation consisted of the 150 patients with the lowest predicted 18-month mortality risks. To obtain the next subpopulation of 150 patients, the 30 patients with the lowest mortality risks were replaced by the 30 patients with the next highest mortality risks. This process was repeated until all patients were included in at least one of the subpopulations. For this study, two STEPPs were created: one with the difference in NMB between care-as-usual and basic support as the outcome and another with the difference in NMB between intensive support and basic support as the outcome. Basic support was selected as the reference category because it was previously shown to be the optimal population-wide strategy [22]. Based on the observed patterns of treatment-predicted risk interaction, a suitable strategy for assigning different DMPs to different risk groups of patients was subsequently identified.

### Quantification of the efficiency gains resulting from the subgroup strategy

To evaluate the optimality of our proposed subgroup strategy, we quantified its efficiency gains as suggested by Coyle et al. [8]. First, the average NMB was evaluated separately per DMP for each of the established risk categories. Subsequently, the average gain in NMB resulting from stratification compared to the best performing population-wide strategy was calculated as $\overline {{{\text{NMB}}}} =\sum\nolimits_{j} {\frac{{\Delta {\text{NM}}{{\text{B}}_j} \times {n_j}}}{N}}$, where $\Delta {\text{NM}}{{\text{B}}_j}$ denotes the difference in average NMB between the proposed treatment for subgroup *j* and the best performing population-wide strategy (basic support in our case), ${n_j}$ denotes the sample size of subgroup *j*, and *N* denotes the sample size of the overall study population. In the previously conducted economic evaluation, it was suggested that the New York Heart Association (NYHA) class, which is a generally used functional classification to describe the severity of HF symptoms, could be a suitable basis for offering different treatments to different subgroups of patient. For comparative purposes, the average gain in NMB resulting from using NYHA class as the stratification basis was computed as well. 95% confidence intervals (CIs) for the efficiency gain estimates were obtained through bootstrapping.

## Results

### Patient-level mortality risk and NMB estimates

The median of the patient-level 18-month all-cause mortality risk estimates was 0.23 (inter-quartile range: 0.14–0.37) with a minimum value of 0.01 and a maximum value of 0.93. Table 1 summarizes the distribution of the patient-level mortality risk and NMB values stratified by the three different DMP groups. It shows that patients from the basic support group had the lowest median mortality risk and the highest median NMB when the willingness-to-pay threshold was set to €20,000. This is consistent with the previously conducted economic evaluation [22] where basic support was found to be the optimal one-size-fits-all strategy.

**Table 1:** Patient-level mortality risk and NMB estimates stratified by the three DMP groups

Patient subgroup (sample size)	Median (IQR) 18-month all-cause mortality risk	Median (IQR) NMB (€)
Care-as-usual (*n* = 339)	0.25 (0.14–0.40)	22,880 (3544–29,012)
Basic support (*n* = 340)	0.22 (0.14–0.35)	24,146 (7253–28,573)
Intensive support (*n* = 344)	0.24 (0.12–0.37)	24,064 (5256–28,208)

*IQR* inter-quartile range

### Exploration of treatment-predicted risk interaction and determination of subgroup strategy

**Fig. 1:**
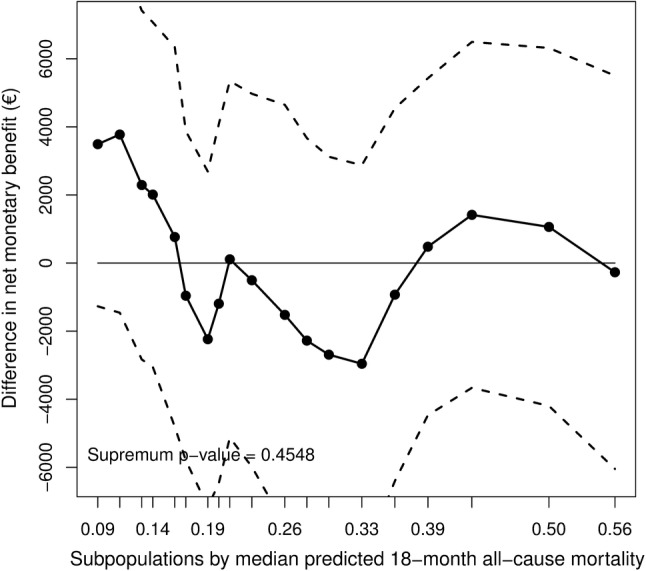
STEPP comparing the difference in NMB between care-as-usual and basic support across different, but overlapping subpopulations with increased mortality risk; a difference in NMB > 0 indicates that care-as-usual is the preferred strategy

**Fig. 2:**
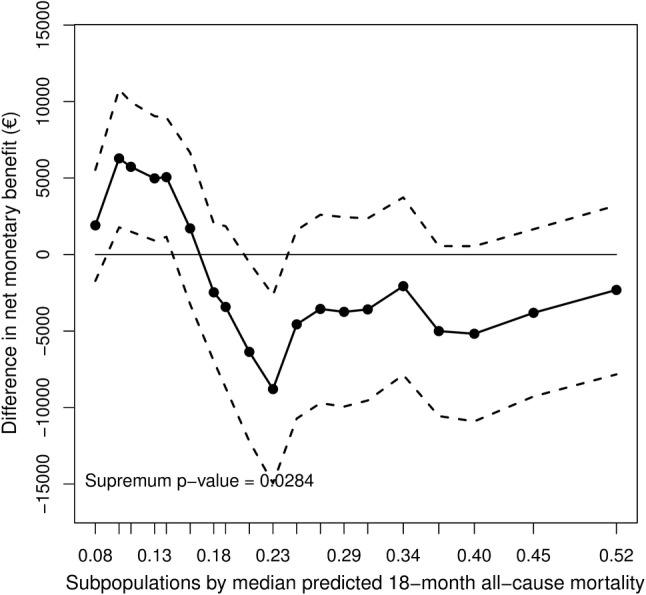
STEPP comparing the difference in NMB between intensive support and basic support across different, but overlapping subpopulations with increased mortality risk; a difference in NMB > 0 indicates that intensive support is the preferred strategy

The estimated difference in NMB across the overlapping patient subpopulations are depicted in Figs. 1 and 2. The pattern of the difference in NMB between care-as-usual and basic support does not suggest clear cutoff points to stratify patients into different risk groups (Fig. 1**)**. In addition, the fact that the treatment–covariate interaction effect was not significant (*p* value > 0.05), suggests that patient heterogeneity did not have a clear impact on the difference in NMB between these two treatments. However, a significant treatment–covariate interaction was found when comparing the difference in NMB between intensive support and basic support (Fig. 2). In addition, the pattern depicted in the plot suggests a risk value of 0.16 to be the zero point at which the difference in NMB between these two treatments starts to change signs. Based on this finding, our proposed subgroup strategy was to assign intensive support to low-risk patients (patients with predicted risk value ≤ 0.16) and basic support to intermediate- to high-risk patients (patients with predicted risk value > 0.16).

### Subgroup cost-effectiveness results

Table 2 depicts the results of the cost-effectiveness analysis within each risk stratum. For the low-risk patients, intensive support was found to be the best performing strategy with the highest amount of NMB, while basic support performed best in the intermediate- to high-risk patients. When NYHA class was used as the stratification basis, basic support was found to be optimal for less severe patients (i.e., those belonging to NYHA class II), while care-as-usual was found to be optimal for severe patients (i.e., those belonging to NYHA class III and IV).

**Table 2:** Results of the cost-effectiveness analysis

Patient subgroup (sample size)	Mean (95% CIs) survival time (days)	Mean (95% CIs) cost (€)	Mean (95% CIs) NMB (€)
*Predicted 18-month mortality*			
Risk ≤ 0.16 (*N* = 321)			
Care-as-usual	521.2 (497.5–543.6)	6151 (3961–8826)	22,389 (19,200–25,101)
Basic support	525.6 (505.2–545.5)	8653 (6117–11,664)	20,127 (17,107–22,844)
Intensive support	557.2 (547.7–562.0)	6213 (4950–7624)	24,307 (22,857–25,577)
Risk > 0.16 (*N* = 702)			
Care-as-usual	428.2 (403.2–451.6)	11,175 (9348–13,226)	12,265 (9791–14,496)
Basic support	454.0 (429.6–480.5)	10,041 (8257–11,935)	14,819 (12,608–17,071)
Intensive support	432.3 (406.1–456.9)	13,155 (11,221–15,142)	10,525 (7935–12,803)
*NYHA class*			
NYHA II (*N* = 513)			
Care-as-usual	481.4 (455.9–504.5)	8955 (6884–11,522)	17,405 (14,318–20,026)
Basic support	506.6 (486.0–528.6)	7170 (5788–8898)	20,570 (18,607–22,250)
Intensive support	505.7 (484.8–527.0)	9099 (7256–11,220)	18,581 (16,087–20,758)
NYHA III and IV (*N* = 495)			
Care-as-usual	428.7 (397.0–462.3)	10,692 (8279–13,206)	12,788 (10,112–15,948)
Basic support	443.7 (414.7–471.1)	11,793 (9435–14,403)	12,507 (9465–15,219)
Intensive support	448.9 (422.2–474.8)	12,462 (10,279–14,779)	12,118 (9304–14,707)

### Quantification of the efficiency gains resulting from the subgroup strategy

Table 3 depicts the average gains in NMB (95% CI) resulting from each subgroup strategy. Both strategies were found to be cost-effective compared to assigning basic support to the whole patient population. However, the subgroup strategy proposed in this study outperformed the one proposed previously, with an average gain in NMB of €1174 (95% CI €− 1146–€3284).

**Table 3:** Average gains in NMB (95% CIs) resulting from each subgroup strategy

Stratification basis	Subgroup strategy	Average (95% CIs) gain in NMB (€)
Predicted 18-month mortality	Intensive support to low-risk group Basic support to intermediate- to high-risk group	1312 (390 to 2346)
NYHA class	Basic support to NYHA II group Care-as-usual to NYHA III and IV group	138 (− 1854 to 2246)

## Discussion

STEPP is a relatively new approach to graphically explore treatment–covariate interaction with limited application in the clinical field [23–26]. By using STEPP to graphically explore treatment–covariate interaction, we found that the difference in NMB between intensive support and basic support varied greatly across different, but overlapping subpopulations defined with respect to increasing levels of predicted 18-month mortality risk. The difference in NMB between care-as-usual and basic support, in contrast, never led to a clear pattern of treatment–covariate interaction. By subsequently selecting the 18-month mortality risk at which the difference in NMB between intensive support and basic support started to change signs as the cutoff to stratify patients into two risk categories, we found that compared to applying basic support to all patients, the use of a stratified approach based on offering intensive support to low-risk patients and basic support to intermediate- to high-risk patients would result in an average gain in NMB of €1312 (95% CI €390–€2346).

Our finding that more intensive multidisciplinary disease management is not beneficial in intermediate- to high-risk patients may seem counterintuitive to some readers, but is consistent with the study conducted by Pulignano et al. [27], who concluded that “most eligible patients for a hospital-based DMP may be those at intermediate risk who are not too sick and not too healthy”. Our STEPP for care-as-usual against basic support suggests that this also holds for the moderate form of disease management that was provided in the COACH study. However, our other STEPP indicates that, compared to basic support, low-risk patients may still benefit from a more intensive form of disease management. Although there is also evidence to suggest that intensive, post-discharge disease management is unnecessary in low-risk patients [28–30], our latter finding is consistent with several previously conducted subgroup analyses. Hebert et al. [31] found that when comparing severe (NYHA class III and IV) and less severe (NYHA class I and II) patients, nurse-led disease management was more likely to be cost-effective in the less severe patients. Similarly, Miller et al. [32], who conducted a model-based evaluation to investigate the lifetime cost-effectiveness of telephonic support for systolic HF patients, obtained a slightly less favorable cost-effectiveness ratio for this intervention after NYHA class I patients were eliminated from their study population. Finally, Goehler et al. [33] found that the median lifetime incremental cost-effectiveness ratio increased with €15,900/quality-adjusted life year (QALY) for male patients and €600/QALY for female patients when the average age of the cohort passing through their model was increased from 55 to 75 years. When combining our results with the findings presented in these previous studies, it seems that the trade-off between a moderate or intensive form of disease management is shown especially in patients at low or intermediate risk who are not too sick to be treated. Patients at high risk, in contrast, do not seem to benefit from a more intense form of multidisciplinary disease management. The question of whether such patients should therefore only be offered a basic form of disease management is an ethical discussion that is beyond the scope of this paper.

In our analysis, we applied a previously developed multivariable risk prediction model to combine the information captured within several covariates into a single prognostic index to represent baseline risk. We subsequently used this index to explore for heterogeneity in treatment effect across different subgroups of patients. Compared to conventional subgroup analysis based on a single prognostic covariate, integrating multiple independent patient characteristics associated with the outcome parameters of interest in a multivariable risk prediction model improves risk stratification [34, 35]. This, in turn, can greatly enhance the statistical power to detect variations in treatment benefit as was shown in a previously conducted simulation study [36]. Moreover, the use of such a multivariable approach avoids the problem of multiple testing, resulting from the need to repeat the subgroup analysis for different individual risk factors. Thus, the chances of obtaining false positive findings are reduced [36, 37].

While treatment-predicted risk interaction can best be assessed on a continuous scale [38], discretization of the predicted risks into two or more ordinal categories becomes essential if we want to use the underlying risk prediction model to guide the selection of therapy. By deriving the cutoff of 0.16 from the treatment effect pattern observed in a STEPP, we were still able to make effective use of the discriminative power of a continuous prognostic index in our quest for an efficient reimbursement policy. This does not hold when applying conventional subgroup analysis based on a single prognostic covariate as we did as part of our previous economic evaluation in this patient population [22]. When quantifying the net benefit gains of one over the other stratification basis, the subgroup strategy proposed in this study was found to outperform the previous one with an average gain in NMB of €1174 (95% CI €− 1146 to €3284).

A limitation of this study is that the cutoff of 0.16 may be specific for the data analyzed in this paper. It was selected by taking into account the pattern of treatment–risk interaction in a single clinical trial. Future research is thus required to determine to what extent this cutoff can also serve as a suitable stratification basis for other studies. Secondly, rather than using an external model (i.e., a risk prediction model developed on another dataset), we used an internally developed risk prediction model to assess the treatment effect across different subpopulations of predicted risk. The validity of this approach was recently assessed by Burke et al. [34], who concluded that “appropriately developed internal models produce relatively unbiased estimates of treatment effect across the spectrum of risk”. In addition, these authors also found that “when estimating treatment effect, internally developed risk models using both treatment arms should, in general, be preferred to models developed on the control population”. As all treatment groups of COACH were included in the development of the COACH risk prediction model, this is exactly the strategy that we have followed in the current paper. Thirdly, because we selected the difference in NMB as the measure of treatment benefit, our results are conditional on the value assumed for the willingness-to-pay threshold. As a first paper to introduce the application of our proposed approach, we only selected a single threshold. For actual decision-making purposes, it would however be recommended to perform sensitivity analysis and repeat the approach for different values of the willingness-to-pay threshold to make sure that the risk-stratified treatment recommendation is robust with respect to the selected threshold value. Another limitation of this study is that the time horizon for the economic evaluation was restricted to the 18-month follow-up period of the COACH study, meaning that cost differences and survival benefits are likely to be underestimated. In future applications of our proposed method, one could therefore consider extrapolating the patient-level cost and survival estimates beyond the range of the trial data by applying more advanced statistical modeling techniques, such as the multi-state modeling approach proposed by Cao et al. [39]. Finally, heterogeneity in individual patient preferences was not considered in our analysis, although it was suggested as being an important factor when developing personalized treatment recommendations [5].

To conclude, the emerging role of health economics in personalized medicine has recently been recognized and is actively discussed [40–45]. To assess how personalized medicine may maximize the net benefits, it is crucial to develop a risk-stratified treatment recommendation [46] to ensure subgroup cost-effectiveness analysis. Recently, value of information analysis was adapted to develop stratified treatment recommendations that maximize net health benefit or NMB [9, 10]. This technique may be useful when a model-based economic evaluation is conducted. Our proposed approach based on STEPP enables the development of stratified treatment recommendations when the economic evaluation is conducted alongside a clinical trial.
